# Best practices for supporting researchers’ mental health in emotionally demanding research across academic and non-academic contexts

**DOI:** 10.1080/17482631.2025.2464380

**Published:** 2025-02-26

**Authors:** Mary L. Quinton, Karen L. Shepherd, Jennifer Cumming, Grace Tidmarsh, Maria R. Dauvermann, Sian L. Griffiths, Sally Reynard, Amanda Skeate, Anita Fernandes, Tasneem Choucair, James Downs, Karen Harrison Dening, Meghan H. McDonough, Lizzie Mitchell, Daniel J. A. Rhind, Charlie Tresadern

**Affiliations:** a School of Sport, Exercise and Rehabilitation Sciences, University of Birmingham, Birmingham, UK; b Institute for Mental Health, University of Birmingham, Birmingham, UK; c School of Psychology, University of Birmingham, Birmingham, UK; d Birmingham Women’s and Children’s NHS Foundation Trust, Birmingham, UK; e Mind, UK; f Project Advisory Group; g Patient Representative, Royal College of Psychiatrists, UK; h Dementia, UK; i Faculty of Kinesiology, University of Calgary, Calgary, Canada; j School of Sport, Exercise and Health Sciences, Loughborough University, Loughborough, UK

**Keywords:** Research culture, psychologically informed, researcher well-being, sensitive topics, emotional labour, participatory action research, deweyan pragmatism, Co-design, bioecological approach, lived experience

## Abstract

**Purpose:**

Researcher mental health in emotionally demanding research (EDR) has been recognized as important, but research to date has often been limited to academic research contexts, qualitative research, or single disciplines. The aim of this study was to identify best practices to promote researchers’ mental health in EDR across academic and non-academic contexts.

**Methods:**

Twenty-six researchers experienced in EDR (aged 33–64) were recruited across sectors and disciplines (e.g. sport psychology, palliative care, conflict resolution). Semi-structured online 2:1 interviews were conducted between October 2023 and January 2024. The co-designed interview guide asked questions on best practices at individual and contextual levels when undertaking EDR. Interviews were analysed through reflexive thematic analysis.

**Results:**

Three themes were generated: (1) the need for a psychologically informed research culture; (2) actions and principles in the immediate research environment; and (3) researcher boundaries with the research, others, and oneself. Underlying mechanisms across themes included tailored, iterative and flexible, and collaborative.

**Conclusions:**

A shift is needed towards a more psychologically informed research culture to support mental health in EDR. Findings have implications for research organizations, conference organizers, and funders as greater resources are needed for researchers in EDR, regardless of method, discipline, or sector.

## Introduction

Researcher mental health in emotionally demanding areas has received increased attention over the past 20 years (Clift et al., 2023; Karcher et al., 2024; Rager, 2005). Emotionally demanding research requires “a tremendous amount of mental, emotional, or physical energy and potentially affects or depletes the researcher’s health or well-being” (Kumar & Cavallaro, 2018, p. 648). Such research consists of unique challenges that can result in negative consequences for the researcher (Dickson-Swift, 2022), including a lack of preparedness (Fenge et al., 2019), vicarious or secondary trauma (Dickson-Swift, 2022), emotional labour (Bergman Blix & Wettergren, 2015), unanticipated disclosures from participants during data collection, and a lack of support throughout the research process (Mallon & Elliott, 2019). Consequently, physical (e.g., digestive and sleep problems), emotional (e.g., crying, guilt) and intellectual (e.g., questioning research identity) consequences have been frequently reported by researchers (Borgstrom & Ellis, 2020; Dickson-Swift, 2022; Rager, 2005). These negative impacts can remain long after the research has concluded (Coles et al., 2014), leading to chronic burnout and change of career choice (Williamson et al., 2020), indicating an urgent need to support the mental health of researchers involved in emotionally demanding research.

A variety of strategies can mitigate the negative impact of emotionally demanding research. Such strategies can be divided into three broad categories; individual-level actions including preparatory checks (e.g., identify safety issues, emotional risks; Brougham et al., 2023; Smillie & Riddell, 2023) and self-care techniques (e.g., journalling, balancing time between sensitive research and activities away from the research; Berger, 2021; Brown, 2023; Loyle & Simoni, 2017), relational strategies (e.g., peer support, debriefs; Fenge et al., 2019; Smillie & Riddell, 2023), and organizational level support (e.g., access to psychological support and costing this into applications; Burrell et al., 2023; Kumar & Cavallaro, 2018). Despite these initial attempts to identify effective strategies, there has been a predominant focus on researchers conducting qualitative (Clift et al., 2023; Dickson-Swift, 2022; Silverio et al., 2022) or discipline-specific research (Batey & Szedlak, 2024; Brougham et al., 2023; Carroll, 2012; Woodthorpe, 2009). There remains a lack of research taking a broader approach to identifying best practices for supporting the mental health of researchers across research methods, disciplines, and contexts, which would facilitate a more in-depth understanding of the nuances involved and identify where tailored support is required.

Attempts to understand the impact of emotionally demanding research on researchers have predominantly focused on those working in academic contexts (Burrell et al., 2023). This focused conceptualization of the “researcher” fails to account for those working primarily in non-academic settings, including individuals in charitable and healthcare sectors, peer researchers, or experts by experience (for a discussion on these terms see Faulkner & Thompson, 2023; Gupta et al., 2023). These researchers can face different challenges, such as lower availability of resources (financially, practically, and emotionally), compared to those in academic settings (Machin et al., 2023). Peer researchers have also reported negative impacts on their mental health when navigating challenges including negotiating their identity, feeling less respected than their academic peers, and experiencing a lack of consideration for structural challenges (Faulkner & Thompson, 2023; Ross et al., 2023). With the recent increase of co-production and participatory research methods, it is important to understand how to best support all researchers’ mental health, regardless of job role and identities, to avoid harm and the further perpetuation of power imbalances that exist through tokenistic and inequitable involvement (Romsland et al., 2019; Ross et al., 2023).

There is an increasing recognition of how the environment within which research takes place can shape and influence researchers’ mental health (Berger, 2021; Mallon & Elliott, 2020; Seifer, 2018). For example, Edelman’s (2023a) Trauma and Resilience Informed Research Principles and Practice (TRIRPP) framework emphasizes the importance of considering how different parts of the research context (culture, institutional and structural) can influence “researcher vulnerability”. Similarly, bioecological theories of human development place the individual at the heart of the immediate and distal systems around them, shifting the focus solely from the individual (i.e., researcher) and emphasizing the interaction between the individual and their context (i.e., person-environment fit) (Bronfenbrenner & Morris, 1998). Despite a range of strategies identified by researchers working in emotionally demanding areas, the feasibility of implementing these strategies will depend on the interaction with the research context (e.g., people and culture), which may not always be conducive to supporting researcher mental health (e.g., due to stigma, lack of resource). To date, however, there is a dearth of empirical research that considers how researcher mental health can be supported across the different levels of the environment and tailored for academic vs. non-academic contexts.

Research culture is an important contextual factor to consider, and “encompasses the behaviours, values, expectations, attitudes and norms of our research communities. It influences researchers’ career paths and determines the way that research is conducted and communicated” (The Royal Society, 2024, p. 1). There is a close relationship between research culture and researcher mental health. Often discussed in a negative light, the challenges of a neoliberal academic culture (i.e., the heavy reliance on success metrics, “publish or perish” culture) are well documented (Skea, 2021). This culture can lead to increased stress and mental health difficulties and a perception of a lack of sufficient workplace wellbeing support and can also lead to poorer research outcomes (Limas et al., 2022; Watson & Turnpenny, 2022; Wellcome Trust, 2020). Importantly, certain sub-groups of researchers are reported to be at greater risk of poorer mental health outcomes, for example, early career researchers, due to the unique challenges faced (e.g., precarity of fixed-term contracts) (Brown, 2023; Canti et al., 2021; Limas et al., 2022; Skea, 2021). Despite the Wellcome Trust (2020) reporting researchers in academia felt significantly more stressed than researchers working in industry, researcher mental health in non-academic contexts remains underexplored. Collectively, this research highlights a need for a shift in research culture that would not only benefit researchers in emotionally demanding research across academia but also those in the third sector and associated relevant parties (e.g., funders, conference organizers). Also, a shift towards understanding what makes for good mental health and the contextual features of those settings supports a more salutogenic and preventative approach aligned with policy and public health theory (Keyes, 2005; Langeland & Vinje, 2022).

To address the aforementioned gaps in the literature, the methodological approach undertaken in this study was participatory action research (PAR), defined as “a collaborative, iterative, often open-ended and unpredictable endeavour, which prioritizes the expertise of those experiencing a social issue [e.g., research negatively impacting mental health, authors own insertion] and uses systematic research methodologies to generate new insights” (Cornish et al., 2023, p. 2). With the exception of Mallon and Elliott (2019, 2020), there is a lack of defined participatory approaches for conceptualizing the impact of emotionally demanding research. As such, the resultant research has a limited audience and impact (e.g., academics who can access journal publications behind paywalls). A PAR approach is required to prioritize action and social change (i.e., a shift in research culture) to ensure researchers’ mental health does not deteriorate due to their occupation. By recognizing the expertise of researchers working healthily within emotionally demanding areas, this approach would include people with a vested interest in creating a change in research culture in a meaningful, authentic, and participatory way. Therefore, the current study included researchers working in academic and non-academic contexts in both an advisory group and participant recruitment to provide depth of understanding on how researchers’ mental health across different contexts is best promoted.

The aim of this study was to identify how to promote researchers’ mental health in emotionally demanding research across academic and non-academic contexts. With the inclusion of an Advisory Group, a qualitative, PAR approach was undertaken to explore and synthesize best practices from world-leading experts across disciplines and research sectors. Therefore, this study makes an original contribution by (1) taking a PAR approach to promote meaningful engagement, (2) conceptualizing the researcher broadly to consider those working in both academic and non-academic contexts, and (3) exploring best practices across multiple disciplines. By doing so, this research enables increased understanding of how to protect researcher mental health across different research contexts and seeks to create a more positive and inclusive research culture.

## Materials and Methods

### Methodological Approach

This research was conducted within a Deweyan pragmatist research paradigm (Dewey, 1938). Starting with problem identification (i.e., researcher mental health being compromised through doing emotionally demanding research), pragmatism is concerned with directing attention to real-world issues and solutions (i.e., establishing best practices in emotionally demanding research). Deweyan pragmatism is not concerned with establishing universal truth, but rather through iterative cycles of reflection and action, “truth” is continually tested through experimental inquiry (Hall, 2013). An essential part of this ongoing inquiry is taking others’ perspectives into account within research communities (Hall, 2013). Thus, the present research aimed to understand best practices through ongoing reflection with an advisory group and participants’ feedback to guide research inquiry (Morgan, 2014).

#### Positionality statement

The lead researcher is an early career academic, with a background in sport and strengths-based psychology and working with young people experiencing disadvantage and complex support needs. Having worked across a range of emotionally demanding research projects, the motivation for the present research stemmed from positive experiences in their mental health being proactively supported (e.g., regular debriefs, access to reflective practice with clinical psychologist), but also frustrations from the lack of support others receive, and learning about actions counterproductive to supporting researchers’ mental health. Through recognizing the expertise of others already exemplifying best practices, and aligned with the pragmatist paradigm, a PAR approach with the inclusion of an advisory group was undertaken.

#### Advisory group

The advisory group consisted of eight people (5 female, 3 male) recruited through adverts, local advisory groups, and purposive sampling due to their experience of best practices within emotionally demanding research. Members varied by career stage, discipline, and research sector. Three meetings took place over the project duration (February 2023 to July 2024), where members were paid for their expertise in line with the payment policy of the charity partner, Mind (an England and Wales-based mental health charity). Ongoing reflection with the advisory group-shaped decisions and the direction of research inquiry (e.g., adding interview questions, interpretation of findings). To be clear, we do not claim this research to be entirely co-produced. Rather, advisory group members consulted and provided specific feedback, but the end decision remained with the lead researcher. This co-design approach was taken due to the level of financial resource available, thus tailoring their involvement accordingly to ensure members were paid appropriately.

#### Recruitment and procedures

A purposive sampling strategy was employed to recruit participants across research disciplines and sectors who had either published manuscripts, guidance, or toolkits on best practices in emotionally demanding research, or led relevant communities of practice. Additional snowball sampling was employed based on suggestions from advisory group members and co-investigators (Patton, 2002). Recruitment and data collection occurred simultaneously, where recruitment stopped when the richness of the data was deemed sufficient to answer the research aims (i.e., information power; Malterud et al., 2016). Following ethical approval from the University of Birmingham’s Science, Technology, Engineering and Mathematics committee (ERN_0847), participants were recruited via email, social media, or through gatekeepers where deemed appropriate (e.g., for peer researchers within mental health charities). Participants were provided with an information letter which outlined their right to withdraw and confidentiality and data storage procedures. Participants were offered a £25 (or international equivalent) e-voucher as a thank you for their time.

#### Participant characteristics

Twenty-six individuals (*M* age = 45 years, *SD* = 8.5) were recruited across disciplines and sectors, including academia, industry, the NHS, the charity sector, and peer researchers with lived experiences related to mental health challenges. To note, research topics were conceptualized as emotionally demanding, but not as “sensitive”, due to previously acknowledged limitations with this term (e.g., what is considered “sensitive” will vary according not just to topic, but also lived experiences; Edelman, 2023b; Mallon & Elliott, 2020). Further demographic information is presented in Table I.

**Table I.** Participant demographics.

Characteristics	n
**Gender**	
Male	5
Female	21
**Ethnicity**	
White	22
Asian or Asian British	1
Black, African, Caribbean, or Black British	1
Other (self-reported as LatinX)	1
**Research experience (years)**	
3–5	2
5–10	6
10–15	4
15–20	8
20–25	4
25+	2
**Main research topic**	
Mental health (across clinical and sport psychology, policy, young people)	10
Forensic psychology	2
Sociolinguistics	2
Social care	2
Neonatal nursing	1
Palliative care	1
Education	1
Social inequalities	1
Death and dying	1
Misogyny and child sexual abuse	1
International security	1
Health psychology	1
Research ethics	1
Loneliness	1

### Data Generation

Pragmatism is not associated with a particular method, but rather aims to answer the research question with the most appropriate method (Feilzer, 2009). In this instance, 2:1 interviews were chosen as most appropriate to understand participants’ expertise. This approach involved two interviewers and a single interviewee, chosen for its ability to allow for more in-depth exploration, enhance rapport development with the participant, and provide support between researchers (Monforte & Úbeda-Colomer, 2021). Following similar procedures as Lévesque et al. (2020), one interviewer led the interview, while the second interviewer took notes to capture non-verbal cues and the interviewers’ observations as part of reflexivity. When not leading the interview, the second interviewer could interject to seek clarification, whilst also noting resources signposted to via the chat function to probe further in-depth responses and understanding.

Interviews were conducted via Microsoft Teams to allow for involvement from diverse geographical locations, and were audio and video recorded. The interview guide was co-designed with advisory group members. Informed by the TRIRPP framework (Edelman, 2023a) and Bronfenbrenner and Morris (1998) Process-Person-Context-Time (PPCT) model, the guide included questions addressing best practices within emotionally demanding research at contextual (e.g., “What can others do to best support researchers’ mental health within these areas?”) and individual levels (e.g., “What can individuals do to protect their own mental health when doing emotionally demanding research?”). Participants were sent an interview guide 1 week in advance to allow time to reflect and prepare notes if desired. Participants were also given flexibility and choice about their interview (e.g., time of day, location, camera on/off). Participants were given scheduled 90 min slots, and interviews lasted between 47 and 87 mins (*M* = 61.9, *SD* = 12.5).

At the beginning of each interview, participants provided informed consent and demographic information through an online form. The semi-structured guide was then followed balanced with participants’ leading of the conversation, with probing questions used to explore responses in more depth. At the conclusion of each interview, participants were asked if they had any further comments before the second interviewer provided a summary of the participants responses and offered, the opportunity to clarify or expand on any points. The automatically generated verbatim transcripts via Microsoft Teams auto-transcribe software were reviewed, errors corrected, and identifiable content removed before sending to participants as part of the member reflection process. Aligned with pragmatism, this process was an important part of the research inquiry, embedding the research community in the cycles of reflection and feedback to generate additional data and insight (Morgan, 2014; Smith & McGannon, 2018).

### Data Analysis

A core tenet of pragmatism is reflexive research practice as part of inquiry (Feilzer, 2009), thus interview data was analysed through reflexive thematic analysis (Braun & Clarke, 2019). Braun and Clarke's (2021) six phases to reflexive thematic analysis were followed, in an iterative rather than linear manner, to develop and refine themes based on reflections from the research community. The two interviewers began by familiarizing themselves with the data, revisiting video recordings, transcripts, and reflexive journals, noting any initial analytic insights. Using NVivo 20 software, the interviewers then independently coded the transcripts with semantic and latent codes, before generating initial themes by identifying shared meaning patterns. The two interviewers then met to review candidate themes, revisiting the data as required to facilitate decision-making. Candidate themes were then presented to the broader research team who served as critical friends, guiding the ongoing analytic process (e.g., noting that candidate themes were more representative of codes). The lead interviewer then developed and reviewed themes using offline methods (post-it notes), revisiting the data and reflexive journal to generate themes and sub-themes. Critical friend meetings with advisory group members and the broader research group provided further insight and alternative perspectives (e.g., ensure the responsibility for researcher mental health is not misinterpreted as being solely the researchers’), where themes were then refined to clarify their central organizing concept and scope.

**Figure 1.**
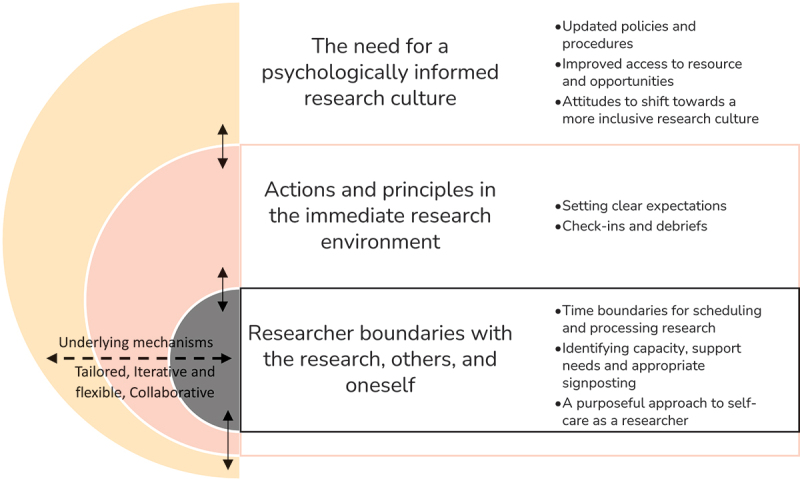
Illustration to show interaction between themes and underlying mechanisms.

As researchers have argued (Braun & Clarke, 2021; Smith & McGannon, 2018), research cannot be solely inductive as reflexivity and theoretical knowledge guide the analytic process. At this point, the lead interviewer took an abductive approach to generate connections between data and theory, going back and forth between the two in alignment with pragmatic inquiry (Feilzer, 2009). Using visual mapping (Braun & Clarke, 2021) and informed by the PPCT model (Bronfenbrenner & Morris, 1998), a thematic figure was produced (Figure 1). Themes and sub-themes were then labelled, defined, and refined to avoid “topic summaries” (Braun & Clarke, 2021), before the final writing-up phase.

Credibility is important within Deweyan pragmatism (Hall, 2013), which was embedded within this research through methodological rigour techniques (e.g., questioning assumptions through member reflections, considering alternative viewpoints through critical friend meetings; Smith & McGannon, 2018). A range of quotes provided throughout the results and supplementary Table I are also provided with the intention of transparency.

#### Researcher mental health

Although the interview content was not anticipated to be “sensitive” as questions were focused on best practices, qualitative research is known to be emotionally demanding (Dickson-Swift, 2022; Silverio et al., 2022). Thus, the researchers chose to proactively identify and implement strategies to look after their own mental health, which included creation of self-care plans, limiting interviews to no more than two per day, post-interview breaks and co-reflection meetings, regular check-ins, and awareness of available resources for support if required.

## Results

Three themes were generated from the data: (1) the need for a psychologically informed research culture; (2) actions and principles in the immediate research environment; and (3) researcher boundaries with the research, others, and oneself. Interactions between themes and underlying mechanisms across themes as illustrated in Figure 1 are also discussed. Quotes are not attributed to specific contexts due to advice from participants and advisory group members to be cautious of “not putting people into boxes”, but the narrative compares and contrasts participants’ reflections across contexts. Further quotes can be found in Supplement Table I.

### Theme 1: The need for a psychologically informed research culture

This theme describes the policies, procedures, and practicalities as part of the broader research environment which are conducive to supporting researcher mental health. Participants discussed the necessity for a psychologically informed, whole systems approach, where researcher mental health (especially within emotionally demanding topics) is acknowledged and actively supported across different organizational levels (e.g., within research team to senior leadership) and formats (e.g., small group meetings to large conferences). As one participant described:

I think as a team we all have a role to support each other, but clearly line managers and then the team leader need particular sensitivities and particular responsibilities. But I think you know that what, what the trauma informed approach is telling us and psychologically informed, those models tell us that it has to be a whole system approach, doesn’t it? You can’t just pick off individuals, it has everything. So all policies that we have to be in the service of being psychologically informed. We can’t have the psychologically informed bit here and then be having demands on people over here and say, oh well that’s different that’s that, it all has to be seen with that lens, and as such all the people that are involved have to work in that in that way as well. (P20)

Importantly, participants discussed the challenges to promote researcher mental health within the context of a negative, neoliberal research context:

But actually no matter how resilient or how much mindfulness somebody does, or how much they choose to jog to work, or you know what, what’s their calorie intake, or don’t drink a lot in the week. All that stuff that actually some of that literature takes you to, in terms of how to cope, is not actually a replacement for decent work, working conditions, proper expectations for managers, you know, none of it. And I think there’s this kind of neoliberal fix, which is individuals can sort it, whereas actually some of it is systemic. (P20)

### Updated policies and procedures for supporting researcher mental health

This sub-theme captures the policies and procedures conducive towards a psychologically informed research culture. The changes of such procedures are likely outside of the control of the researcher and the immediate support network, and therefore ultimately depend on the level of investment and resource from the organization. Participants described a need for either existing policies and procedures to be updated to consider researcher mental health, or the creation of such policies where they did not exist.

Participants discussed the integration of researcher mental health into procedural ethics applications, *“You know when we’re going through ethics, it’s just as important to look at the researcher’s mental health and well-being and what they’ve got as much as it is for you know, those that they’re looking to safeguard.”* (P1). Ideally, ethics committees would be aware of relevant resources to signpost towards for researchers in emotionally demanding topics:

Like it would be nice to say, see a situation where there’s enough financial provision for support so that a committee could say, absolutely, go ahead, but we’d like you to at least have an introductory session with, you know, the counselling services that are available just so that you know somebody’s name and you know what it would be like and you’re aware of what’s there, you know, that sort of thing, or that you attend the monthly community of practice around mental wellbeing or something like that. (P26)

Although procedural ethics is embedded in academia, researchers in other contexts struggled when these processes were not formalized, “*I’ve had the question asked to me, so do we need ethics for this bit of work that we’re doing? And it’s because it’s not part of the culture … I have been in situations where that’s felt very uncomfortable”*. (P24). In these contexts, participants discussed how they would use their prior academic training to inform their approach, “*it is baked into your sort of DNA as a researcher. And so you have to kind of decide what form that’s going to take, you know, are you going to kind of just write your own ethics protocol?”* (P24).

Another area relevant to both contexts was safeguarding protocols. By the researcher having a clear understanding of safeguarding protocols of the organization and/or fieldwork context, researchers would immediately know the recommended course of action in scenarios affecting someone’s safety (e.g., participant disclosures that require escalation). Participants discussed that the researcher would then be less likely to ruminate on these difficult situations, thus their mental health would be less likely to be compromised compared to if they were less aware of such policies:

I think part of part of protecting people’s well-being as researchers is that they understand the nature of confidentiality and consent and what it is that they not only can share, but actually have an obligation to share. If they need to raise a safeguarding alert. And I think that can give people confidence to be able to manage what can be very triggering and quite difficult moments in the interaction. (P20)

Related to these protocols, possession of a work mobile phone and email address was perceived as important for managing safeguarding concerns appropriately.

### Improved access to resource and opportunities

This sub-theme encapsulates the optimum level of resource and opportunity available to the researcher and their research team. Such resource is likely provided by the immediate organization, but could also be provided by external sources (e.g., international communities of practice and training courses).

An area of resource discussed across both contexts for researchers’ conducting emotionally demanding research was the provision of and access to mental health support. Although access to services as part of organizational support (e.g., employee assistance programmes; EAPs) was perceived as more beneficial than no support, participants discussed that EAPs were not always the most useful type of support due to professionals not knowing the specific research context and the limited number of sessions available. Rather, access to reflective practice or 1:1 sessions (e.g., with a clinical psychologist) were seen as a more useful, tailored form of support. Participants discussed that this related to the broader research culture as the provision of this support should be costed into funding applications and should be available for the duration of the project, so has consequences for funders and organizations to be receptive to this when stating eligibility criteria of what can be costed:

It’s not like you’re going to have a crisis and then you need to access the support. It’s there right from the very beginning so that you can talk about how you feel going into a project… I just think it’s a really important thing to have access to that is being paid for by the project. But also is not seen as a kind of fall back if you’re struggling, it should just be part of the support that you have to do a difficult job. And it needs to be framed that way. I think it’s often framed wrong. (P10)

Where this type of mental health resource had larger financial considerations, access to peer support networks was commonly discussed as a beneficial and a lower cost resource. These could be informal (e.g., within research groups) or more structured (e.g., communities of practice for researchers doing emotionally demanding research across the world). As exemplified by this quote, the purpose of these networks was not to fix problems but often served as validation that researching such areas can be emotionally demanding, “ *… having a community of people who they can, who are also working on sensitive things is helpful because even if you’re not talking about your own work directly, at least you’re working in a space where people understand.”* (P10)

Mentoring schemes were also discussed as helpful for more tailored support from those with greater experience, who were perceived as role models:

I think it was more like mentoring around it. And the good bit of that, which is why I’m raising it, was it felt very helpful to have an identified person that I could talk to about what I was struggling with and about the research and how to handle it, because they were very used to handling sensitive topics. (P26)

The final part of this sub-theme relates to the need for increased training opportunities. These were discussed in the context of integrating researcher mental health in emotionally demanding research into staff inductions, particularly in relation to limiting unnecessary exposure of difficult content to others. However, the discussion was mainly directed towards those who support researchers. This related to psychologically or trauma informed training, as discussed in the context for academic supervisors below:

When people do research with, you know, in labs with chemicals or biological materials, they have to get a certificate that says I have been trained in working with hazardous materials and that qualifies them to do that research. I think if you’re going to be in this area and working with survivors, for the participants sake, I think everybody should have some sort of trauma informed training and probably some self-care training and for the researcher’s sake I would love to see that kind of thing mandated. (P6)

### Attitudes to shift towards a more inclusive research culture

This sub-theme represents a need for a shift in attitudes around researcher mental health and subsequent strategies implemented. Although there were exceptions, participants indicated that generally non-academic contexts embodied a more inclusive and supportive research culture, as outlined by a former non-academic researcher:

It’s just kind of research culture really. It’s, there’s this culture of like if you’re not constantly working then you’re going to fail your PhD, or you’re not going to get that publication, you’re not. So I think firstly research culture, and that that can come from, so I’m not, I know I’m being quite generalizable in that it sounds like every lab is like this. It’s not at all, and if you have a good PI [principal investigator] who creates that space to say, look, it is OK to go and do this. So here at [non-academic organisation], we just have a culture of it’s fine to go and take those breaks if you need to. And there’s also kind of like internal barriers as well in that you can, if you’re anything like me, you can be told these things, but sometimes because you’ve had that [negative] research culture ingrained into you, there’s that kind of like, oh, I must keep working, must keep working. So it’s kind of like those two levels, the systemic higher level, but also the internal, oh, you’re fine, just get on with things, kind of thing. (P21)

Compared to contrasting attitudes on academia:

Well, I mean, self-care and academia. You know they’re not often seen as things that go hand in hand. I mean that to some extent is a stereotype of the area. And it’s a shame. It’s a really unfortunate one, but it’s, you know, the general idea is that academics generally don’t have time for this. You know, they’re incredibly busy. But, I think to some extent, you know, that’s part of the problem. We should change that and actually make self-care a key element of, you know, supervisors’ lives and then they model the best practise for their students. (P1)

Participants discussed the need to “normalise the conversation” that mental health can be affected by the research:

I think there’s a danger of pathologizing. You know, as human beings, we’re supposed to experience all a range of emotions. So there’s something about, again, I suppose that’s about the normalizing. So it’s about responding to it, but without pathologizing it, I suppose that feels important. (P26)

However, this was difficult due to the stigma existing within research culture, *“In my context, it’s the taboo we should not talk about our emotions there, since it is professional, so you know, keep professional and personal separated”* (P5), and:

First of all, normalising it, or rather, destigmatizing it by saying this is a thing that can happen. So acknowledging that from the outset, I think already reduces stigma and makes conversation possible. I think that’s a really important strategy that can be overlooked (P26)

Related to this destigmatization, participants were passionate that a shift away from a neoliberal agenda with the onus on individual resilience was needed, *“it’s not about being the toughest croc on the rock, you know you can’t just expect people to be resilient”* (P7). Instead, a shift in the broader research culture to be more supportive would improve attitudes and experiences for researchers. Although the below quote refers to an academic context, this participant was comparing to the non-academic context they now worked within. This quote also emphasizes the interaction required between different systems (Figure 1), as this participant describes that for the researcher to benefit, their manager should receive training, which requires resource and investment from the organization:

I could talk for ages on this because it frustrates me that a lot of universities put the onus on the individual. It’s like, oh, do this training on resilience. I hate that word resilience, resilience training. Because it’s like, oh, you need to be more resilient to put up with this research culture, even though you’re researching sensitive topics, you just need to have more resilience. Do self-care, go for a walk. When actually I feel like the training should be for line managers and PIs to really get to grips with changing that kind of systemic research culture of, and I think this is across the board. Again, this is not just researchers researching sensitive topics, so that should be across the board, but it should be a particular case for PI’s working in sensitive topics and the whole lab is working in sensitive topics. (P21)

A specific approach mentioned to improve attitudes was the implementation of content notes (also discussed as trigger or content warnings). Participants mainly discussed their experiences at conferences, where best practices included providing advanced notice, “So giving trigger warnings much earlier you know … they had outside each of the breakout rooms, they had what the breakout room was about, and also trigger warnings” (P26), and presentation alterations, “So I’ve gone to conferences that you know, [sensitive] content, where they posted a quote up but not read it out loud and allow people to read it at their own pace.” (P25)

A key reason why content notes were seen as inclusive was for those with lived experience of the research content. One participant (P26) discussed the typical approach of providing notice immediately prior to starting the presentation. However, without advanced notice (e.g., in an event programme booklet), those who then wish to leave the room cannot do so without “outing themselves”, as exemplified below:

But actually that’s problematic when you’re in a big room or you’re with people that you don’t know because again, it’s saying if you’d like to out yourself as a trauma survivor or as triggered, then just leave the room. You know, there’s no way, if you’ve given the invitation, that you keep your bum on the seat unless you’re upset, then you’re basically saying in order to look after yourself, you’re going to have to out yourself and that I have real problems with.

To mitigate this, more general approaches can be integrated alongside content notes:

When we invite people to take a break or take a breather, let’s just do that as a general thing. Let’s just make that a general mental wellbeing thing. If you need to stretch your legs, if you need to get up and use the loo, if you need to go and get a glass of water during this, just do. We don’t have to say, if you’re distressed or if you’re triggered, or if this thing happened to you too.

### Theme 2: Actions and principles in the immediate research environment

This theme represents the proactive behaviours shared between the researcher and those in their immediate support network, and the underpinning principles to facilitate such behaviours. Participants described this immediate environment to be heavily influential in supporting researcher mental health.

### Setting clear expectations

This sub-theme involved being clear on the details of involvement and ways of working through proactive planning. This communication was often the responsibility of the supervisor or project lead, but aspects also related to a collaborative approach between them and the researcher.

Participants discussed the importance of setting expectations about what the research would entail, such as the types of methods and forms of data, and stating this clearly from the recruitment stage:

When we’re recruiting people as researchers, as research assistants or postdoc researchers, it’s being really open and honest, at the beginning, about what the nature of the research is and what that person might be exposed to so they can make an informed decision as to whether that’s the right project for them (P9)

Once employed, the ways of working between the researcher and immediate support network should then be clarified. A key starting point participants stated was creating a researcher “mental health” or “wellness” plan. This plan was seen as the foundation of taking a proactive approach to identify effective strategies and support that may be required, as exemplified by this quote in the context of working with peer researchers:

We are really good at providing a dynamic, flexible and ongoing support to our young people, so that includes things like wellness plans, so getting young people to reflect around what sort of supports work for them and how we can help them during their involvement. How to identify if they’re starting to feel distressed and what we can do to help them. (P16)

Participants emphasized that these plans should be reviewed throughout the project and protected time was essential to prioritize these conversations, “*I’m really trying to make sure that time is protected … so with my PhD students, for example, we’ve set up like 6 monthly meetings, specifically going through … their well-being action plan for in the workplace*.” (P12). Setting clear expectations was also discussed for the end of the research project. Especially when researching “controversial” topics, it was seen as the supervisors’ responsibility to discuss best practices on dissemination for safeguarding the researcher and participants:

And then, you know, coming out the other side in terms of disseminating. And this comes from the trauma informed work as well, that they would have done in preparation and that’s umm, the ethics of sharing these results is so important, and also what talking about it, what effect it may have on the researcher by disseminating this, and the ethical dilemmas around that. You know, not to sensationalise, but to be true to the survivors’ disclosures, you know you have to be true to the data, but how do you do that in an ethical way. And working with the participants I have found is really helpful for safeguarding the researcher, so going back to the participant and say, you know, what are you comfortable sharing and how can I be of help in being true to your stories. You know that you’ve generously shared with without you know it being performative or sensationalising etc. so it sounds like it’s for the participant and it very much is, but that kind of interaction discussion is also part of safeguarding the researcher. (P6)

Once the research or the researchers’ contract has ended, it was also perceived as important for researchers’ mental health to manage these endings explicitly:

Well, actually the one area we haven’t talked about is sort of the equivalent of an exit interview … once the student or research personnel is finished with a project, let’s sit down, talk about what went well in the project, what could have been done differently, but also, for the for the researcher, what went well? What didn’t? Why? Why not? What? What could be done differently? And so that that’s an important kind of monitoring and assessment piece, that you’re sort of doing informally throughout the project? But making it an intentional piece at the end of a project, I think it’s really important. (P22)

### Check-ins and debriefs

This sub-theme entails the organization of regular check-ins and debriefs throughout the project. This was spoken about at individual and team levels. For example, for more general check-ins, this participant outlined:

So we’ll have times where sometimes you know, we’ll have a meeting and we’ll go through the things and then, there’ll always be a little bit of the meeting which is just checking in, ‘is everybody OK? “Does anybody want to talk about anything? If it’s not appropriate to talk about in this space, let’s book a face-to-face space where we can do that”, so we do a lot of checking in actually. (P10)

Although general check-ins were important, specific meetings throughout collecting and analysing data in emotionally demanding research were essential:

Just kind of good briefing and I think definitely having a briefing afterwards, with all supervision, you know, and just to kind of talk about anything that is lingering and perhaps it can be over a longer period, not just straight after. Sometimes people can be triggered, sort of after some time as opposed to it being straight after the research itself. (P14)

#### Theme 3: Researcher boundaries with the research, others, and oneself

This theme describes understanding researchers’ limits for research engagement and the establishment of healthy boundaries through reflection and clear communication.

### Time boundaries for scheduling and processing research

The first sub-theme was the necessity for time boundaries to protect researchers’ mental health. Participants discussed practical implications of limiting the time spent on the research. This could be done proactively, such as when scheduling data collection (e.g., limiting number of interviews per day), or analysis (e.g., limiting amount per week), as outlined in the quotes below:

… to make sure that you have breaks from that field work, you know, I mean, I’m falling into that as well that we end up scheduling interviews back-to-back because that’s, you know, that’s the everyday experience, isn’t it? But that means that you’ve been listening to different people talking about different, you know, potentially really difficult topics hour after hour after hour. It wears you out, so you know, trying to protect yourself in that respect a little bit more, that you really have got some down time, that you you’re able to take a break from that field work that’s really important. (P2)

We only have them code difficult data two days per week as part of a full time week. So kind of bordering out, you know, if there are particular aspects that are very sensitive and you know exposure to them is difficult, kind of you know bracketing or like you know not expecting people to do it on a full-time basis. (P13)

Time boundaries were also crucial for processing the research at all stages. Some participants spoke about allowing time to prepare or “pre-resting time” before data collection or analysis, “*So before I do online meetings I mentally, it’s always 10 to 15 minutes before I start that I’m like OK, let’s just like decompress*” (P8). Whereas others focused on needing sufficient time to process the data and decompress after data collection or analysis, *“I’m gonna take the appropriate amount of time after a very sensitive and very emotional, perhaps a very traumatic account”* (P23). The use of reflective tools such as journalling facilitated processing, ”*I permit myself to have the time to process what I’ve done. You know, I always keep a kind of reflective diary in terms of projects that I’m ongoing now.”* (P1)

Despite these time boundaries being considered as essential for staying mentally healthy, participants spoke about the challenges of maintaining these boundaries due to funders and commissioners’ strict timelines within both academic (first quote) and non-academic (second quote) contexts:

… timelines, funding these may serve as a barrier as well … I think it’s students, that are maybe in a more vulnerable position when they just know they need to finish this project and they’re hoping to kind of sweep maybe this emotional burden or that they’re struggling under the rug because they need to meet that next deadline (P23)

… someone’s paying for a piece of research and the decisions taken by other people about what the timescale is, and if that’s not realistic, then that that just ends up putting you under a huge amount of pressure, been in that situation before. Actually, thinking about in terms of mental health that that has been one of the most stressful kind of scenarios that I’ve come up against. So, you need, do you need, you just need a comfortable amount of time, and a recognition as well of how long analysis takes. (P24)

### Identifying capacity, support needs and appropriate signposting

This sub-theme referred to researchers’ personal capacity for research engagement, which may relate to one’s own lived or living experiences, and the associated avenues of support. Importantly, researchers’ lived experience or preferences do not equate to avoiding the topic area completely, but rather there may be certain methods, literature, or forms of data to limit engagement with and maintain those boundaries.

I’m very aware of what I can and can’t do. I generally have a rule, for example, that I don’t work on imagery of any kind if I can avoid it. I prefer not to work on audio if I can, if I can avoid it, just because those kinds of mediums of data are more difficult… So, I think as a researcher, it’s knowing yourself and being boundaried and sticking to that is really important. (P10)

Although self-awareness is required by the researcher to identify their limits, it was also deemed the supervisors’ or managers’ responsibility to understand who they are working with by understanding specific learning and communication styles and personal experiences where relevant to the research.

For me obviously as a manager I would support people and try to make adaptations and have the flexibility to accommodate people’s different mental health [needs] and issues in relation to neurodiversity for example, all those things that kind of come together as a bit of a package (P20)

This responsibility also included awareness of appropriate sources of support, and knowing when to signpost to these sources as part of setting and maintaining clear boundaries in their professional relationship with the researcher, as outlined by this participant, “*There’s difficulties that come up that are about the research itself, and there’s difficulties that come up that are about how the research triggers your own personal issues. And maybe that’s the distinction where you refer somebody for external support*”. (P22)

### A purposeful approach to self-care as a researcher

This sub-theme included deliberate strategies or behaviours that were perceived as beneficial towards maintaining mental and physical health. These behaviours were not specific to the research context, but rather more general self-care strategies such as “*exercise, get out in nature, connect with people, eat well, try and get to sleep at a reasonable time*”. The physical removal of oneself away from the research environment underpinned the effectiveness of these strategies, as illustrated below:

I also build into my day a lot of walks, so I tend to have lunchtime walks. And those walks are really, really useful to just clear your head…I tend to listen to music, other people may be listening to podcasts, whatever. But again, it gets you away from the immediacy of what’s going on. (P2)

### Underlying mechanisms

It was apparent that there were core mechanisms that underpinned best practices for researcher mental health in emotionally demanding research, which spanned across themes (Figure 1) and are discussed below.

### Tailored

Participants emphasized that supporting researcher mental health is not a “one size fits all” approach but rather each researcher will be different, thus a tailored approach is needed that recognizes who the researcher is, who they have around them, and the research culture they work within:

Of course, some people like talking about things in groups, some people don’t, and I think what I’ve really learned is that … people will need different kinds of support. What we have to make clear is that that support is available to everybody in the team, but that doesn’t mean that everybody will access the same kind of things. (P20)

### Iterative and flexible

Regularly revisiting the effectiveness of strategies for researchers’ mental health throughout the research process was essential:

What they’re doing in terms of self-care, do we need to adjust their self-care plan and those sorts of discussions, and very often, and this is an important thing as well is, I think to safeguard researchers, we need to go into this knowing that it’s very much an emergent design. You know, you’re gonna decide. I just don’t have it in me to listen to another interview. I’m not gonna even schedule one until I’m ready. Or, you know, shifting plans. So having that flexibility, that agility in terms of the research plan is really important. (P6)

This iterative approach was not only necessary because of research content but also because what can be “emotionally demanding” may change over the course of the project. Therefore, there is a need to be proactive in responding to new or changing circumstances (e.g., unanticipated life events, required changes to data collection method), *“I think sometimes people don’t realise that what they’re working on is something that might become emotionally challenging, and it might not be emotionally challenging all of the time. It might become so because something else has happened.”* (P10)

### Collaborative

Unanimously, participants indicated the onus should not solely be on the individual, but rather a shared responsibility between researcher, their manager (or supervisor), and the policies, people and organizations in the broader research culture for appropriately supporting mental health. As illustrated in Figure 1, the themes are represented in a matryoshka format which recognizes this interaction between the researcher and their immediate and distal environments. These interactions are further exemplified in this quote describing available sources of support:

So, supervisors really need to be kind of in tune with those individual level resources, but also have an awareness of when they need to escalate, maybe to other resources beyond themselves. Because again, I don’t think the impetus lies on the supervisor to be, you know, a counsellor and a full-scale mental health practitioner when that, when that is required, they need to have the pathways or be able to point to the resources for this individual. And then there needs to be those institutional level supports. So, if the supervisor is going to be supported or the researcher in early career researcher, I think in institutions need to know where to go when they are maybe struggling are their mental health supports. Are there employee resources for these career researchers that they’re able to say, look I’m being impacted by my research work, and that that isn’t a foreign concept for at that institutional level. So, I see it as being very, very layered and awareness is really needed at those different levels that there can be this impact that mental health can be affected by the type of research work that we do. (P23)

## Discussion

The aim of this study was to identify best practices for researcher mental health in emotionally demanding research. Although research has investigated strategies to mitigate the negative impact of such research (Burrell et al., 2023; Kumar & Cavallaro, 2018; Smillie & Riddell, 2023), to our knowledge, this is the first study to date to (1) take a PAR approach to promote meaningful engagement, and (2) conceptualize the researcher broadly to consider those working in both academic and non-academic contexts. This research also extends the literature by exploring best practices across multiple disciplines, rather than being method (e.g., qualitative) or discipline (e.g., psychology) specific. Altogether, this research supports the essential role of the research context and culture (Berger, 2021; Mallon et al., 2020), without which best practices cannot be achieved. Importantly, this finding was evident across both academic and non-academic contexts.

Engaging non-academic in addition to academic researchers conducting emotionally demanding research generated new insights to inform practice to support researcher mental health. Specifically, participants discussed that academic contexts typically had greater resources to support researcher mental health than non-academic contexts, such as greater financial support, access to training, and procedural ethics policies. Non-academic contexts were seen as conducive to supporting researcher mental health through how self-care was embodied and implemented (e.g., breaks encouraged, well-being plans more commonly integrated, reflective practice), compared to less tailored approaches in academia (e.g., provision of training on self-care and resilience, access to generic mental health support). This finding is supported by research which found that industry researchers felt less stressed on average than their academic counterparts (Wellcome Trust, 2020), which may be explained by the more tailored support discussed above. The resultant implications for best practices indicate that more tailored approaches are required to align with a more conducive approach to supporting researcher mental health. Furthermore, co-learning between sectors and more collaborative research could help offset these challenges in the different sectors.

Although the strengths and challenges of each context were discussed, it was also clear through the diversity across participants that researchers do not fit into “boxes” of academic or non-academic. This was evidenced by numerous participants who spanned sectors, had moved from one context to the other and thus varied in their experiences. Furthermore, it was evident that lived or living experience of mental health difficulties was not limited to peer researchers. The importance of recognizing different intersections is an important conceptual contribution to challenge harmful stereotypes that “those wearing the hat of professional or academic are not the ‘sort of people’ to have experienced traumatizing adversities” (Edelman, 2023b, p. 2), or that those in peer researcher roles are not as knowledgeable as academics (Gupta, 2024). By broadly conceptualizing who are researchers, these nuances have important implications for future practice and policy to support researchers’ mental health (both within emotionally demanding research and more generally) to ensure a more equitable approach to mental health provision in research. It is important to recognize all researchers contributing to a project, especially in participatory approaches where existing hierarchies may marginalize those with lived experiences and those from minority backgrounds (Faulkner & Thompson, 2023).

One of the most prominent themes of this research was the need for a psychologically informed research culture. Psychologically informed approaches, as part of the psychologically informed environment (PIE) model, are concerned with understanding the emotional and psychological needs of people and tailoring interactions and support accordingly (Johnson & Haigh, 2010). When participants discussed best practices, numerous suggestions related back to the PIE model, such as reflective practice, in-house psychologists, managing relationships, and staff training (Keats et al., 2012). Where PIE originates from the homelessness sector (e.g., Skeate & Templeton, 2024), closely linked to psychologically informed approaches, there has been increased attention towards trauma-informed approaches in research, which support the needs of those who have experienced trauma (Edelman, 2023b). Researchers have argued for the implementation of trauma-informed approaches for researchers in emotionally demanding research to avoid the risk of re-traumatization (Berger, 2021; Dickson-Swift, 2022; Edelman, 2023b). However, researchers involved in emotionally demanding research may require support regardless of their previous exposure to trauma, or whether the research is “traumatising”. Therefore, a psychologically informed approach promotes the broader emotional support needs of researchers, moving beyond neoliberal tendencies of individual responsibility (e.g., becoming more resilient) and putting the onus on research organizations and funders to provide appropriate resource and infrastructure (Edelman, 2023a), acknowledging the structural aspect of emotionally demanding research and its consequences.

One relevant principle of psychologically informed approaches for those supporting these researchers is the elastic tolerance approach (Boag, 2020), whereby the need to “perform” (i.e., the expectation of the researcher being healthy enough to do their job) should be balanced with a compassionate approach (i.e., compassion for why someone might struggle with emotionally demanding research and appropriate flexibility given). A shift towards more psychologically informed research cultures would not only have positive implications for researcher mental health but could also indirectly contribute to a healthier work force (e.g., less sick days, lower burnout and stress, and less career attrition; Williamson et al., 2020). In the USA, an emerging body of literature is investigating how institutional cultures and practices influence individuals’ physical, mental and social health (e.g., the Healthy Campus movement, Posa Flynn et al., 2018; Seifer, 2018). Future research would benefit from exploring the role of higher education institutions in health promotion for staff, students, and their local communities, especially within emotionally demanding contexts (e.g., leveraging existing resources towards tailored mental health support).

As part of a psychologically informed research culture, participants emphatically advocated for a more proactive and inclusive approach to researcher mental health. Importantly, these discussions did not pathologize, but rather emphasized that emotional responses to emotionally demanding research were normal. Thus, being prepared and proactive is important for promoting researcher mental health in emotionally demanding work (Burrell et al., 2023; Fenge et al., 2019). Proactive practices, such as the inclusion of content notes, also align with equality, diversity and inclusion policies where mental illness (e.g., post-traumatic stress disorder) may be considered as a protected characteristic if classified as a disability. Therefore, where there is a legal obligation to provide proactive reasonable adjustments (Carey & Travis, 2020), this has implications not only for organizations (e.g., in research and teaching) but also for conferences and events involving emotionally demanding content (Edelman, 2023b). In the words of one participant, an inclusive research culture should ensure that “people are not in any way disadvantaged in terms of their mental health by working in the team and the research that they do”. Although the feasibility of implementing the best practices discussed in this research will vary, we encourage all involved in research to reflect on how even small changes can be made to implement more psychologically informed practice.

### Strengths, limitations and future research

The strengths and original contributions of this research include the PAR approach and advisory group integration to address the problem. Little research investigating the impact of emotionally demanding research on mental health has undertaken a multidisciplinary approach (for exceptions see Fenge et al., 2019; Mallon & Elliott, 2019). The current research also explored best practices across sectors and methodologies to further understand the nuances of this complex issue. Finally, a methodological strength included sending the interview guide 1 week prior to the interview. Participants frequently fed back that they found this useful to reflect and prepare answers if desired. Therefore, we recommend this as an inclusive approach for future research, which may be particularly suitable for neurodiverse participants.

There are some noteworthy limitations which are important to contextualize this research. First, whilst efforts were made to recruit a diverse sample, participants worked predominantly in Westernized contexts and were of White self-reported ethnicity. Mental health stigma varies across different cultures (Abdullah & Brown, 2011), thus future research should explore perspectives from non-Westernized contexts and minoritized groups, including discourse-focused approaches to understand how unhelpful cultural norms are upheld and how researchers’ social identities influence their experience and need for specific strategies. Second, the sample predominantly included those who identified as female. Although this was representative of the literature on those who have published within this area, future research should integrate additional perspectives who can contribute to the conversation (e.g., those who identify as gender non-conforming). Third, these best practice suggestions will not be feasible for all contexts. Currently, there is no literature outlining the feasibility of the implementation of strategies for researchers in emotionally demanding areas. Aligned with Deweyan pragmatism, where inquiry outcomes are referred to as “warranted assertions” which must then be tested in real-world settings (Hall, 2013), future research would benefit from exploring the extent to which strategies need to be tailored and the feasibility of strategies to understand what works for whom, in what contexts, and why, as part of the overall person—environment fit (Bronfenbrenner & Morris, 1998). Finally, due to the well-documented challenges of working with a neoliberal research culture (Limas et al., 2022), future research would benefit from exploring the feasibility of such strategies for all researchers’ mental health regardless of working within emotionally demanding areas, especially for those already at greater risk of poorer mental health outcomes (e.g., early career researchers; Brown, 2023; Skea, 2021).

In conclusion, this research supports the essential role of a psychologically informed research culture, without which best practices to support mental health in emotionally demanding research can be impeded. This research also provided conceptual contributions to the literature by defining the researcher broadly across academic and non-academic contexts and recommends that co-learning between sectors could help offset challenges faced by the different sectors. Altogether, these findings have implications for research organizations but also those within the broader research culture, such as conference organizers and funders, as greater provision and resource is needed to shift towards a more inclusive culture for all researchers in emotionally demanding research, regardless of method, discipline, or sector.

## Supplementary Material

Supplement table 1.docx

## Data Availability

Data associated with the paper is not publicly available as we did not ask for participants’ consent to publicly share the raw data. However, supplementary Table S1 provides a further example quotes for transparency.
