# Supplementary material for: Best practices for supporting researchers’ mental health in emotionally demanding research across academic and non-academic contexts
Source: Int J Qual Stud Health Well-being. 2025 Feb 26;20(1):2464380. doi: 10.1080/17482631.2025.2464380 (PMC11866648; doi:10.1080/17482631.2025.2464380)
Supplement: Supplement table 1.docx [file ZQHW_A_2464380_SM8902.docx]

**Supplement Table 1.** Themes and sub-themes with additional example quotes.

| **Theme** | **Sub-theme** | **Example quote** |
| --- | --- | --- |
| **The need for a psychologically informed research culture** |  | “But actually the whole context in which we do the research can also be difficult as well. And I don't just mean the data collection. I mean that in the course of meetings …And how do we do trauma informed work in a trauma informed way. And by that I mean any kind of meetings or training events or anything around the subject of being trauma informed. But I think that applies more widely as well. So, for example, I talk about let's start with the assumption that everybody in the meeting is traumatised because you don't harm somebody by being careful about content, but you can harm somebody by not being careful about content. So you can do things like having, um, almost in your terms of reference, but like, maybe more, more live than that, because whoever looks at those right? It's just to have some ground rules about we don't go into a great deal of detail about things that have happened to us in this context.” (P26)  “And I do think we need culture change. I do think that, you know, we've got it focused all the wrong way round. We're so focused on outputs and funding and numbers and metrics that we need to turn it on its head and actually put health and well-being at the centre and then build everything else around that because I think happy researchers, all of that other stuff will come, but I think unhappy researchers you might get your metrics, but when that researcher is burnt out in a few years’ time, they're they're not going to be any good for anyone, and particularly for their own mental health, I think that's everything.” (P1) |
|  | Updated policies and procedures for supporting researcher mental health | “I think it's having a robust [researcher safeguarding] policy and understanding the [field work] settings’ organisational policy. So it's not about having your own research institution policy. It's having a real knowledge of the setting you're going into and having those communication channels very clearly established.” (P7)  “So there is the safeguarding, obviously, for the research participant, which is absolutely primary concern, but similarly at the same time, I think there's how that relates to individuals’ safety in their own, their own ability to, some people need to know exactly what to do. If they're, if they're, if they're told, you know that somebody is if somebody discloses something, as indeed people very often do, that it's important that they know what to do with that information and then how to find support for themselves. Because I think that is quite... I mean, I know that when I was a much, much more junior researcher, somebody told me something and I was quite plagued by it for a long time. I didn't know what to do with it. I didn't know how to process it. I didn't know if I should tell anybody, you know, all that all that stuff.” (P20) |
|  | Improved access to resource and opportunities | “Yeah. I think if if they're not aware that there are some of those system level supports for themselves, that's obviously a problem. So if they aren't aware that they have employee assistance or that they're student support services, that's problematic. If there are resources out there and they just aren't aware to be able to connect in with them, I often times I think maybe those resources aren't in place or they're not tailored to the type of mental health support that might be necessary for somebody who is being impacted by their research work. It's more generic, especially when I think of the student services level things it's it's it's fairly generalized and so if you came in and said I've just read you know, 50 interviews of someone's accounts of [variety of emotionally demanding topics] all these awful things, that somebody has just read, I don't know that a generic counselling service may be prepared for prepared for that or or, you know, the appropriate response. So that's another limitation I might see that they they are aware of the services they seek out and those services may not be appropriate.” (P23)  “I've had informal chats with colleagues not on the same project who have come to me talking about the experiences that they've had, for example, witnessing distress. And again, there's no formal structure around this. We're not part of the same team. What we are is colleagues who kind of cross into the friend boundary. So sometimes having a complete outsider with experience of witnessing distress, supporting a participant in a distressing situation, et cetera, but no skin in the game. You know, delivering on the project or meeting deadlines or anything else has no impact whatsoever. That seems to be important from experience.” (P17) |
|  | Attitudes to shift towards a more inclusive research culture | “But with staff, I think there's even it, it blows my mind because I have my own lived experience of mental illness and this we and a lot of people at our organisation would, but we don't talk about it. And and I think it's getting better, it'll get better, but we yeah, we don't have those structures or policies in place that it will that enable those conversations. And maybe that's different in other teams, I can't, but certainly in our team that's not really, that's not really the culture, and it's a very supportive culture, but it's it's we don't, yeah, it's just not something we do.” (P16)  “But there is a but there is also a huge culture change required I think, because I think most of the academics I know will not, to me, but I don't think it's me, I think it's just in general, will not admit to vulnerability. They will not actually, because it's such a competitive environment, you know, you, you you have to sort of keep your cards close to your chest. So if someone was really struggling, would they actually tell someone?” (P15)  “So for example for me, I I managed my mental health in various ways, one of which is that I regularly see a psychotherapist, people at work know that I don't get to work until 20 past nine on a Wednesday morning, because I see my therapist on Wednesday morning. You know, I I kind of, it's private therapy, I can afford to pay for that. I do it, it's successful. I am old enough and kind of confident enough to be able to talk about that in a way which is which normalises it. I know that for other people in the team, you know, it could be different. They may not have that ability to that confidence, to normalise it, but they may still need to come in late on a Tuesday, and if they say I can't get here till 10 on a Tuesday, then I'll just have to... I think there's an element in which I just sucked that up and I accept it, not everybody has to has to be out about it” (P20)  “I really dislike the idea of, so I know how I wouldn't define it, is stuff around about like resilience and stuff, which has been trotted out quite, quite a lot in relation to this material. Because I think that that places a lot of the onus on the individual researcher to deal with whatever problems that this research might cause and takes the onus away from institutions to put in place the, you know, support that might be needed. So I think it's, so I think resilience, you know, isn’t, for me at least, isn’t the right way to kind of conceptualize it because it places too much responsibility on an individual researcher who might not necessarily have the systems and processes and networks around about them, that I think universities and, you know, research agencies, agencies, should be embedding, you know, whether that's through training or workshops or, you know, mentoring or counselling or whatever it is.” (P25)  “Like one of my bugbears is you sit down, you put your arse on the seat, whether it's virtual or real, and then somebody goes this is the trigger warning that we're going to be discussing this and this and this. And it's like, oh wow, great. So now again, back to empowerment, my choice is out myself by getting up and leaving. Or sit it out. That's not really a, it's a Hobson's choice right?” (P26) |
| **Actions and principles in the immediate research environment** |  |  |
|  | Setting clear expectations | “...that should be in the recruitment. So when I applied for one of the jobs I have at the moment, I had a conversation with [name redacted] and she explained to me what format the data would be in, and I knew that before I applied for the job” (P10) |
|  | Check-ins and debriefs | “For me, it was really important as a as you know in my earlier research areas, particularly to have that support network and someone to check in with. You know, we often build into ethics that particularly if you're doing sort of off-site interviews or interviews in homes or you know, licenced premises or anywhere in public spheres. You know, I'd often check in with my supervisor 'I'm here now’, ‘I'm safely home now'. This is good, but it's more than that. It's like.' OK, well, how did it go'? You know, let's have a let's have a debrief. Let's actually talk about, you know what they discussed and how you felt about that. That's really, really important. And I do that quite often with my own students. I found that incredibly, incredibly valuable.” (P1)  “And so making sure that there is constant conversation throughout the process that there's check in's. That you know myself as a researcher, I also feel very responsible, and I think when I emailed you about what, like, talk to my students because I feel very responsible for them as well, and I think that's the other aspect that we often forget is that, you know, when we're when we're a researcher and then we're a supervisor and we have students that are going out and doing this work, there has to be a, you know, a scheduled time to check in and to make sure that that everyone's doing OK.” (P18) |
| **Researcher boundaries with the research, others, and oneself** |  |  |
|  | Time boundaries for scheduling and processing research | “And I think there was something there about the volume of interviews they were doing in a day, so sometimes they were doing 3 interviews a day. The more you do, the more tiring it is, and the more you need that check in and support. And I think there is a sweet spot of not doing more than four a day because it's it's overwhelming. So there's a volume of how much you can expect a human to hear. How much distress you can expect to human to process, and that's entirely individual.” (P7)  “It frustrates me seeing funders being like, oh yeah, we want to change research culture or we want significant lived experience component, and considering the finance aspect of it, but not the time aspect of it. Their deadlines are still back-to-back, and they're still during the summer holidays or Christmas holidays, which doesn't help when you've got childcare” (P21) |
|  | Identifying capacity, support needs and appropriate signposting | “But yeah, just make sure that you that you are aware of your own.... the topics that maybe you might find fall short on if there's something specific that you really just don't feel comfortable discussing” (P3)  “And I think having an awareness of your own trigger points is probably useful because that then, then you can in sort of take advance mitigating action, and I mean if you know that something is not good for your mental health, then to the extent possibly avoid it or you know, I'd always tell my colleagues, go and talk to me as your line manager if there's a certain issue that I can then address.” (P19)  “You pretty much got to know when you're beat, there will be cases and there will be things where you just think I can't read that and it's given yourself permission to go, nope I'm not touching that. But you know, there's areas of my work and I just think, no, I'm not going near that it because it will have too much of an effect on me.” (P11)  “I guess a lot of the time and you know if it was a peer support researcher, I I think it would be very important for them to really look at their own lived experience, what they've been triggered by in the past. But if it's a researcher who is a clinical, you know psychologist or a consultant psychiatrist, they may have done it a million times and not be that phased, so it's really, it’s really I think individual. But I think it it just goes back to, I think them knowing themselves and perhaps knowing their boundaries and their limits.” (P14)  “So I would say that I think if there is not sufficient distance or support, it is harmful for people to embark on that kind of research. That being said, all of my research is with lived experience researchers and co-researchers, and it's hugely rich and helpful. So part of it's about the support you offer in the team, but part of it is also about people's readiness to research. I've also, I remember having a big debate with a colleague who was doing eating disorders research from that sort of research in their own experience of eating disorder, but hadn't been in services for some time. And they felt very strongly that that perspective gave them an insight into it, which I could completely see. And I agree with, I'm very keen on co-production and lived experience research, but I'm also mindful that these things are not neutral and they can be very harmful unless they're properly and robustly supported and unless that person has some element of distance from the thing that the object of study, because otherwise you turn yourself inside out trying to find an answer, and if it's very confronting to your own identity, I think that can be quite negative.” (P7) |
|  | A purposeful approach to self-care as a researcher | “I do believe in doing activities that are away from the research work. So I myself I'm constantly engaging in all sorts of creative practises with creative writing with painting, drawing... I'm not necessarily good at anything, but that's not the point. The point is that I've got time to kind of wind down and switch off.” (P2)  “Making sure that you you do the things in life that that bring you joy, you know outside of work, those kind of things.” (P4) |
| **Underlying mechanisms** |  |  |
|  | Tailored | “I think good safeguarding is probably a number of different strategies and support systems that are in place to help protect researchers from, not only working in this space, but then the heaviness of it and ensuring that researchers have their own sort of toolkit to use in in the ways in which they feel and experience the work that they do.” (P18)  “You know we all do the writing and all of that and actually we may find out that the writing isn't working for us and that I don't know audio recording or voice recording are working better working better for us. But unless you've tried it out for yourself, you won't know. So it's not just like you say, you know, it's it's having different tools at your disposal, but also playing with them and to see see whether you like them and and if you do great, stick with them. And if you don't, OK try it another time. If you've tried it a third time and it still doesn't work for you, forget about it. Do something else.” (P2) |
|  | Iterative and flexible | “And then we also have discussions about what works for them in terms of self-care, whether it's exercise or music or, you know, talking with friends, whatever that looks like so that they have a self-care plan in place before they start their data collection. Umm. Then during the data collection as their supervisor, I need to be in touch with them a lot. And again, not only in terms of the research angle, but in terms of how the researcher is doing personally. How they're responding, what sense they're making of what they're hearing”. (P6) |
|  | Collaborative | “Yeah, I do feel like there is an element of self-awareness. Mental health can be very individual. You need to know yourself very well, but I also think there's a risk with putting a lot of the burden or the, you know, the responsibility to take care of mental health on just the individual. And so there's a bit of needing to be a self-aware and needing to know what maybe might trigger you emotionally or what your sources of strength or resilience are, but you may need some obviously more than just that individual level support” (P23)  “And I'm particularly critical of the 'oh they're having a crisis, end them to the Employees Assistance program', which has been mentioned to me about some of my, one of my students. Because the informal support options hadn't been, it's almost like we're gonna farm that out, that's difficult and challenging, and it might be, but you have a conversation with someone about what they need and where they are. And then you might need to refer them to professional support, but I think sometimes there's a tendency to just run straight to the professional support option, and if you are supervising a student or a researcher who's funded in a difficult topic you should expect that some of what you will have to do with that, that person is to support them emotionally, not professionally, necessarily, but just in the day to day kind of what's coming up on a challenging day, for example, being available to have that supportive conversation. And if the problem persists, then good safeguarding is to get professional support, but I don't think it's the first port of call.” (P22)  “So when we talk about a self-care plan, that includes a discussion about either whether you know, whether to, I encourage them to think about whether external support would be helpful, and I talk about the importance of often for some having independent support and that you know in in clinical psychology for example, this is just the way it's done. We haven't made it a norm in the [topic] world, but there's lots to be learned from that model. And so, given the conflicts of interest that students will inevitably have with me, I think it's really important that we discuss who is going to be, you know, their sounding board. And they may have someone in place, but if not, there are lots of people that that I can link them with through the university who can be that independent kind of support, and so my role is not to engage in sort of counselling or therapeutic work, but just check in and if they're not, if I can, if I see they're struggling and they're not getting external support, I revisit that discussion and also revisit the research plan in terms of, you know, maybe we should hold off on the next interview for a little bit. So it is a fine line because I want to know how they're doing, and that requires, you know, setting up a rapport that enables them to say, you know, I'm struggling, without and in turn, I can't be kind of getting into the counselling or therapeutic role, but I I do like to to keep a check on them and be the conduit for other resources. I guess that's the way I just put it.” (P6) |
